# Association of changes in the use of board-certified critical care intensivists with mortality outcomes for trauma patients at a well-established level I urban trauma center

**DOI:** 10.1186/1752-2897-6-3

**Published:** 2012-03-06

**Authors:** Diana Petitti, Vicki Bennett, Charles Kung Chao Hu

**Affiliations:** 1 Center for Health Information and Research, Arizona and the Division of Trauma Care Services, Scottsdale Healthcare Osborn Medical Center, Arizona State University, Phoenix, USA; 2 Department of Biomedical Informatics, Arizona State University, 502 E. Monroe Street, Phoenix, AZ 85004, USA; 3 Division of Trauma Services, Scottsdale Healthcare Osborn Medical Center, 7400 E. Osborn Rd, Scottsdale, AZ 85251, USA; 4 Trauma & Surgical Critical Care, Scottsdale Healthcare Osborn Medical Center, 7400 E. Osborn Rd, Scottsdale, AZ 85251, USA

## Abstract

**Background:**

An intensivist-directed Intensive Care Unit is a closed-model unit in which a physician formally trained in critical care plays a leadership role in patient management. In the last decade, there has been a move toward closed Intensive Care Units. The purpose of this evaluation was to assess the association of changes in the use of intensivists to a closed-model with mortality outcomes in injured patients seen in a long-established urban Level I Trauma Center.

**Methods:**

This analysis used data from the Scottsdale Healthcare Osborn Medical Center trauma registry from January 1, 2002-December 31, 2008. Mortality prior to hospital discharge was compared in the pre-intensivist (intensivists were not employed and did not provide care), partial intensivist (intensivists were employed and provided care during some Intensive Care Unit shifts) and full-time intensivist (intensivists were employed and provided care in the Intensive Care Unit full time) periods. Multiple logistic regression analysis was used to estimate odds ratios for mortality adjusting for patient characteristics and injury severity for the partial intensivist and full-time intensivist periods compared with the pre-intensivist period.

**Results:**

Of 18,918 patients, 365 (1.9%) died before hospital discharge. After adjustment for demographic factors and injury severity score, for all patients, odds ratios comparing the partial intensivist and full-intensivist periods with the pre-intensivist period were 0.84 (95% confidence interval 0.64-1.11) and 0.99 (95% confidence interval 0.69-1.41). In patients with an injury severity score 16-24, the adjusted OR for death was 0.20 (95% CI 0.07-0.58) comparing the partial-intensivist with the pre-intensivist period and 0.30 (95% CI 0.11-0.88) comparing the full-time intensivist period with the pre-intensivist period. For patients age 65 + years, compared with the pre-intensivist period, odds ratio were 0.51 (95% confidence interval 0.31-0.84) and 0.61 (95% confidence interval 0.32-1.16) for the partial and full-time intensivist periods respectively.

**Conclusions:**

In our setting, a change to a closed Intensive Care Unit model was associated with improved mortality outcomes in patients with less severe injuries and patients age 65+ years.

## Introduction

There is convincing evidence that in-hospital mortality outcomes are better for injured patients cared for in Level I Trauma Centers than non-trauma centers [1]. Evidence is limited about the association of other specific staff patterns with outcomes in Level I Trauma Centers [2-4].

Patients in an open Intensive Care Unit (ICU) are managed by independently working physicians who are not generally trained in critical care. An intensivist-directed ICU is a closed-model unit in which a physician formally trained in critical care plays a leadership role in patient management. In the last decade, there has been a move toward closed ICUs. Evidence from a 2002 systematic review and meta-analysis by Provonost et al. [5] provided the initial impetus for this move, which was accelerated by the decision by the Leapfrog Group to make ICU staffing with intensivists one of the four focus areas for improvement in hospital safety.

Because many seriously injured patients are admitted to an ICU, the association of patterns of ICU staffing with outcomes for these patients is of interest. In 2006, Nathens et al. reported the results of an analysis of data from a 68-center prospective cohort study of trauma patients, the National Study on the Costs and Outcomes of Trauma (NSCOT), that showed a lower relative risk of in-hospital mortality following severe injury in hospitals with intensivist-model ICUs compared with hospitals with "open" ICUs (RR 0.78; 95% confidence interval 0.58-1.04) [6]. In 2009, Lettieri, Shah and Greenburg reported improved mortality outcomes in an intensivist directed ICU in a combat zone, where about 40% of admissions were for injuries [7].

In 2010, Lee, Rogers and Horst [8] reported the results of an evaluation comparing outcomes for trauma patients managed in a Level II community hospital trauma program before and after introduction of a model for providing ICU care that relied on the closed ICU approach in which dedicated trauma intensivists provided ICU care 24 hours per day, 365 days per year. No significant differences were found in mortality outcomes or total hospital days, but ventilator days, ICU days, and number of medical consults were significantly lower and days to tracheostomy significantly shorter in the period after introduction of the ICU critical care intenstivist model.

The present evaluation took advantage of naturally occurring changes in the use of board-certified critical care intensivists at a long-established, urban Level I Trauma Center to assess the association of changes in the use of intensivisits with mortality outcomes in injured patients.

## Methods

### Setting

Scottsdale Healthcare Osborn (SHCO) Medical Center is a 334-bed acute care hospital and functions as the only trauma center in the eastern valley of Maricopa County. The Trauma Center was first designated as a Level I center by the state of Arizona in the early 1980's. In October 2008, it was verified by the American College of Surgeons as a Level I Trauma Center. The Trauma Center is located centrally in Scottsdale, Arizona and served a population of about 1-2 million during the period time covered by this evaluation.

### Review

The analysis was carried out using deidentified data. It followed local guidelines for ethics committee review of studies based solely on deidentified data, which classify the study as exempt from ethics committee review.

### Data source

The source of data for this analysis was the SHCO Trauma Registry. The analysis was based on data about patients included in the registry from January 1, 2002 through December 31, 2008. Although trauma registry data extend back to 1995, the analysis was limited to data collected in 2002 and later due to data quality and completeness. For the variables used in the analysis except Abbreviated Injury Score and injured body part, there were no missing values in 2002 and later.

**Figure 1:**
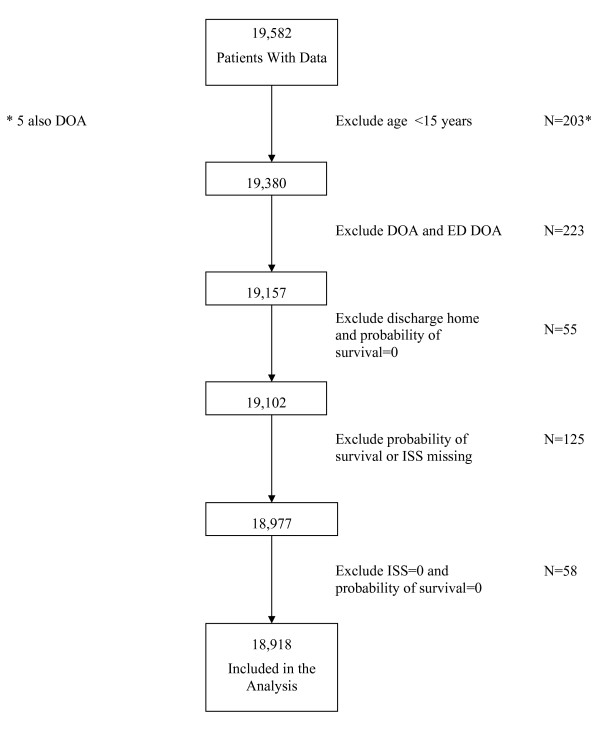
**Flow of patient records into analysis**.

Information was available on 19,582 patients seen during the period from January 1, 2002-December 31, 2008. Because the SCHO trauma center is not designated as a pediatric trauma center, patients whose age was less than 15 years (N = 203) were excluded from the analysis. Also excluded were patients who were dead on arrival at the trauma center (n = 223); patients with an unknown probability of survival or unknown injury severity scores (N = 125); patients discharged home with a probability of survival of zero (N = 55); and patients not discharged home with injury severity scores of zero and a probability of survival of zero (n = 58). The latter two exclusions were made because the data appeared to be due to errors that could not be corrected without consulting the original medical record. Figure 1 depicts the flow of patients into the analysis.

### Use of board-certified critical care intensivists

Prior to October 2005, board-certified critical care intensivists were not employed at SHCO to take care of trauma patients. Board certified critical care intensivists were employed to care for SHCO trauma patients on a limited basis starting in October 2005. Their involvement in the care of trauma patients in the intensive care unit was increased in April 2006. Starting in January 2008, trauma and medical intensivists who were all board-certified provided collaborative care to trauma patients in the intensive care unit full time. This analysis was done comparing mortality outcomes between the three periods defined as follows: a pre-intensivist period (January 1, 2002-September 1, 2005); a partial intensivist period (October 1, 2005-December 31, 2007); and a full-time intensivist period (January 1, 2008-December 31, 2008).

### Outcome

The outcome examined in this analysis was death before discharge from SHCO among trauma patients who were alive when first seen in the trauma center.

### Analysis

The characteristics of patients and their injuries were summarized by period using counts and percents and, for continuously distributed variables, using means. Associations between period and patient and injury characteristics were tested for statistical significance using the chi-square statistic for categorical variables and analysis of variance for continuous variables.

The first step in the analysis was calculation of crude (unadjusted) odds ratios (OR) and 95% confidence intervals (CI) for the association of patient and injury characteristics and period with mortality. Multiple logistic regression analysis was used to estimate the OR and 95% CI for the association of period with death adjusting simultaneously for age and other patient and injury characteristics.

The association between period and mortality was assessed separately in patients with high injury severity scores [9] defined in three subgroups: 16-25, 25-34, and 35-75. The cut-points for this analysis were chosen to be consistent with a sub-group analysis of trauma outcomes data published in 2009 by McKenney et al. [4]. For injury severity scores less than 16, the number of deaths was small overall (N = 32) and for each period; thus, analysis by period was not done in the subgroup of patients with injury severity scores less than 16. For the other three injury severity score subgroups, crude mortality rates were calculated and the statistical significance of the association of death rate with period was tested using the chi-square statistic. Multiple logistic regression was used to estimate ORs and 95% CIs for death by period within each of three subgroups of injury severity scores adjusting only for age and sex. Inclusion of other variables shown in Table 1 in these subgroup analyses did not change the OR estimate by more than 10%.

**Table 1:** Description of the characteristics of patients and their injuries for the pre-intensivist, partial intensivist, and full-time intensivist periods

	Pre-intensivist Period		Partial Intensivist Period		Full-time Intenstivist Period		
		
Characteristic	N = 11,399		N = 5,540		N = 1,979		P-Value*
Age, mean, (SD)	37.2	(17.8)	39.7	(18.8)	42.6	(19.6)	< .001
Died, N, (%)	191	(1.7)	122	(2.2)	52	(2.6)	.004
Male, N, (%)	7,496	(65.8)	3,856	(69.6)	1,403	(70.9)	< .001
Race/Ethnicity, N, (%)							< .001
Hispanic	2,191	(19.2)	1,041	(18.8)	285	(14.4)	
African American	313	(2.8)	161	(2.9)	48	(2.4)	
American Indian	738	(6.5)	360	(6.5)	175	(8.8)	
Asian	97	(0.9)	65	(1.2)	17	(0.9)	
Other/Unknown	146	(1.3)	91	(1.6)	17	(0.9)	
White, non-Hispanic	7,914	(69.4)	3,822	(69.0)	1,437	(72.6)	
Body Part Injured, N, (%)							< .001
Head or neck	4,398	(38.6)	2,140	(38.7)	773	(39.2)	
Face	524	(4.6)	213	(3.9)	84	(4.3)	
Chest	747	(6.6)	572	(10.3)	346	(17.5)	
Abdomen, pelvic contents	538	(4.7)	353	(6.4)	161	(8.2)	
Extremities, pelvic girdle	1,655	(14.5)	1,245	(22.5)	450	(22.8)	
External**	3,533	(31.0)	1,012	(18.3)	160	(8.1)	

Some percentages may not sum to 100.0 because of rounding

* P values are based on the chi-square statistic for categorical variables and analysis of variance for continuous variable comparing the three periods

†14 patients had missing data for injured body part

** lacerations, contusions, abrasions, burns

The association between period and mortality was also assessed separately in patients age 65+ years adjusting for sex and injury severity score. Inclusion of other variables shown in Table 1 in this analysis did not change the OR estimate in this subgroup by more than 10%.

Model fit was assessed based on the Hosmer Lemeshow test [10].

A P value less than 0.05 (2 tailed) was considered significant. All statistical analyses were conducted using SAS software version 9.1 (SAS Institute Inc., Cary, NC).

## Results

Of the 18,918 patients remaining after exclusions, 365 patients (1.9%) died before hospital discharge. Table 1 shows the characteristics of these patients and their injuries by period. There were statistically significant associations between all of the patient and injury characteristics and period (all P's < .05). Of particular note are the lower mean age of patients in the pre-intensivist (37.2 years) compared with the full-time intensivist period (42.6 years) and the higher percentage of patients whose injured body part was categorized as "external," a category that includes lacerations, contusions, abrasions, and burns.

A high proportion of patients had missing Abbreviated Injury Scale (AIS) scores. The percentage of patients with missing AIS scores varied by period. In the pre-intensivist period, data on AIS score were missing for 17.9% of patients. The large amount of missing data on AIS score made it impossible to use the AIS score as either an adjustment or stratification variable in the analysis.

Table 2 ORs and 95% CIs for death adjusting for age and all of the variables in the table. After adjusting for age and all of the other variables in the table, the OR for death was 0.84 (95% CI 0.64-1.11) in the partial-intensivist period compared with the pre-intensivist period and 0.99 (95% CI 0.69-1.41) in the full-time intensivist period compared with the pre-intensivist period. The 95% CI for both ORs include 1.0 and neither is statistically significant (both P's ≥ 0.05). The difference in adjusted ORs for death comparing the full-time period(0.99) and the partial intensivist period (0.84) also were not statistically significantly different (P > 0.05).

**Table 2:** Adjusted odds ratios and 95% confidence intervals (CI) for death for patient and injury characteristics and period

	Adjusted for All Variables in the Table		
Characteristic	Odds Ratio	(95% CI)	P Value
Age	1.03	(1.03-1.04)	< .001
Sex			
Male	1.24	(0.95-1.62)	.12
Female	1.00	(referent)	--
Race/Ethnicity			
Hispanic	0.72	(0.50-1.06)	.10
African American	0.96	(0.36-2.57)	.94
American Indian	1.49	(0.87-2.55)	.14
Asian	1.53	(0.46-5.08)	.48
Other/Unknown	0.79	(0.24-2.65)	.71
White, non-Hispanic	1.00	(referent)	--
Body Part Mainly Injured			
Head or neck	2.12	(0.85-5.29)	
Face	1.00	(referent)	--
Chest	0.78	(0.29-2.07)	
Abdomen, pelvic contents	1.73	(0.63-4.78)	
Extremities, pelvic girdle	0.58	(0.21-1.65)	
External	0.93	(0.32-2.73)	
Injury Severity Score	1.14	(1.13-1.15)	< .001
Period			
Pre-intensivist	1.00	(referent)	--
Partial intensivist	0.84	(0.64-1.11)	.22
Full-time intensivist	0.99	(0.69-1.41.)	.95

Table 3 shows death rates and adjusted ORs and 95% CIs in the pre-intensivist, partial intensivist, and full-time intensivist periods in subgroups of patients defined according to injury severity score and in the subgroup of patients age 65+ years. In patients with an injury severity score 16-24, the adjusted OR for death was 0.20 (95% CI 0.07-0.58) comparing the partial-intensivist with the pre-intensivist period and 0.30 (95% CI 0.11-0.88) comparing the full-time intensivist period with the pre-intensivist period. Both adjusted ORs were statistically significantly different from 1.00 (both Ps < 0.05). There was no statistically

**Table 3:** For subgroups of patients defined by injury severity score and age, number of deaths, death rates and adjusted odds ratios and 95% confidence intervals (CI) for death by period

	Deaths Number	Patients Number	% Died	Adjusted Odds Ratio	(95% CI)	P Value
Injury Severity Score 16-24						
Pre-intensivist period	30	1,000	3.0	1.00*	(referent)	
Partial intensivist						
period	5	718	0.7	0.20	(0.07-0.58)	.001
Full-time intensivist						
period	4	313	1.3	0.30	(0.11-0.88)	.03
All periods	39	2031	1.9	----	----	
Injury Severity Score 25-34						
Pre-intensivist period	78	434	18.0	1.00*	(referent)	
Partial intensivist						
period	55	335	16.4	0.85	(0.58-1.26)	.42
Full-time intensivist						
period	29	136	21.3	1.13	(0.70-1.86)	.60
All periods	162	905	17.9	----	----	
Injury Severity Score 35-75						
Pre-intensivist period	64	195	32.8	1.00*	(referent)	
Partial intensivist						
period	52	146	35.6	1.12	(0.71-1.76)	.62
Full-time intensivist						
period	16	54	29.6	0.87	(0.45-1.68)	.68
All periods	132	395	33.4	----	----	
Age 65+ Years						
Pre-intensivist period	62	1108	5.6	1.00	(referent)	
Partial intensivist						
period	33	712	4.6	0.51	(0.31-0.84)	.009
Full-time intensivist						
period	14	308	4.6	0.61	(0.32-1.16)	.13
All periods	109	2128	5.1	----	----	

* Adjusted for age and sex

†Adjusted for sex and injury severity score

significant difference in adjusted ORs for death comparing the full-time intensivist period and the partial intensivist period in other injury severity subgroups (all Ps 0.05). For patients age 65 + years, the OR for death was 0.51 (95% CI 0.31-0.84) comparing the partial with the pre-intensivist period and 0.61 (95% CI 0.32-1.16) comparing the full intensivist with pre-intensivist period. Only the OR comparing the partial intensivist period with the pre-intensivist period was statistically significant (P < 0.05).

## Discussion

This study found no association of changes in the employment of board-certified critical care intensivists to provide care to trauma patients in the intensive care unit with an increase or decrease in patient mortality overall. After adjustment, mortality in the subgroup of patients with injury severity scores of 16-24, the least severely injured patients, was statistically significantly lower comparing the partial-intensivist and the full-time intensivist periods with the pre-intensivist periods. In the subgroup of patients' age 65+ years, mortality was also significantly lower in the partial intensivist period compared with the pre-intensivist period but mortality was not statistically significantly lower comparing the full-intensivist period with the pre-intensivist period.

In an analysis of data from the National Study on the Costs and Outcomes of Trauma (NSCOT), Nathens et al. reported mortality outcomes for trauma patients managed in hospitals with intensivist staffed ICUs were better than in open ICUs [6]. In the study by Nathens et al., the mortality outcome difference for trauma patients managed in hospitals with closed ICUs was larger for older (age > 55 years) patients. Our study provides support for the contention that institutions that use intensivists for ICU care affect mortality outcomes more in older trauma patients.

Just as in a similar evaluation by Lee, Rogers and Horst [8] there was no decrease in overall mortality after the change to greater use of intensivists in this setting. When the changes in practice were made, the SCHO trauma center was already well-established. Some of the practices that are believed to mediate better outcomes for trauma patients managed in hospitals with intensivist-run ICUs, such as collaborative team care and use of protocols and guidelines [11], may already have been implemented in this setting at the time of the change in practice.

We do not have an explanation for our finding of significantly lower mortality in the partial and full-time intensivist periods in patients in the injury severity category defined by ISS scores of 16-24 (less severe injury). A number of subgroup analyses were done and the finding may be due to chance.

The results of the study should be interpreted recognizing the difficulties that arise when trying to evaluate the effect of changes in workforce on outcomes. The number of deaths among patients cared for at the SHC trauma center was small overall, and the study had limited power to detect true effects of changes in the use of intensivists with mortality overall. Critical intensivists might be expected to have their greatest impact on the outcomes of patients who were admitted to the ICU. The rules for collecting data in the early period of the study did not permit identification of patients who were admitted to the ICU. An analysis limited to patients admitted to the ICU might have revealed an association with outcome that is different than for all trauma patients. Data from 664 patients were excluded from the analysis. The effect of these exclusions on conclusions is not known and this is a further limitation.

The statistically significant changes over time in mean age and the distribution of patients according to race/ethnicity probably reflect changes in the demographics of the area served by the trauma center. The change in the distribution of injured body part is a result of policy changes in the criteria for receiving care in the trauma center. There was a striking decrease in the number and proportion of patients in the category for the injured body part that has the label "External," a category that includes lacerations, contusions, abrasions, and burns. These patients were increasingly "triaged" to the emergency department over time. This change probably explains the increase in mean ISS over time. The increase in the proportion of patients who were male may also be a result of the decrease in the proportion of patients seen in the trauma center with these kinds of injuries. We have attempted to account for the changes in patient and injury characteristics by doing multivariate analysis, but the possibility that the statistical adjustment did not fully account for changes in patient and injury characteristics cannot be ruled out.

Mortality in patients seen at the SHCO trauma center appeared to be lower in the categories of ISS that were used in the subgroup analysis compared with other recent published data and with published data from the National Trauma Data Bank (NTDB). McKenney et al. reported mortality rates for 2006-2008 of 2.85%, 23.0%, and 42.2% in trauma patients with ISSs of 16-24, 25-34, and 35-75, respectively, cared for at the University of Miami, Ryder Trauma Center at Jackson Memorial Hospital [4]. Published benchmark mortality rates from the NTDB as presented by Shafi et al. are 7%, 29%, and 54% in trauma patients with ISSs of 16- 24, 25-34, and 35-75, respectively [12].

The differences between the mortality rates observed for the SHCO trauma center and other centers and the NTDB benchmark data may reflect exclusions made in this analysis. It is also possible that the low mortality in patients managed at this trauma center in the period covered by the evaluation is, as discussed above, because some of the practices that are believed to mediate better outcomes for trauma patients managed in hospitals with intensivist-run ICUs [11] had been implemented prior to the exclusive use of critical care intensivists.

Mortality is a crude measure of trauma outcome and effects of the availability of changes in the use of intensivists on other important outcomes of care are not ruled out by this evaluation. Lee, Rogers, and Horst [8] observed significantly lower ventilator days, ICU days, and number of medical consults and significantly shorter days to tracheostomy for trauma patients in a pre/post evaluation of introduction of trauma ICU intensivists to a Level II community hospital trauma program. Length of stay was not examined in our evaluation because data were missing for a large number of patients and the missing data problem was not considered amenable to correction. Data about ventilator days and medical consults was not collected uniformly across the periods of the study and also could not be examined.

Continuing to improve the quality of care for trauma patients and other patients with high acuity is a priority in the United States and elsewhere. The closed ICU is one strategy that aims to improve outcomes in such patients. In our setting, the change to this staffing model was associated with better mortality outcomes in a subgroup of less severely injured patients and in patients age 65+ years. Future research should examine the relationship of use of intensivists with length of stay, cost and patient-centered outcomes.

## Competing interests

The authors declare that they have no competing interests.

## Authors' contributions

DP and VB participated in the design of the study. DP conducted the analysis of the data. DP, VB, and CH participated in interpretation of the analysis results and in drafting the manuscript. All authors read and approved the final manuscript.
